# Guanidinoacetic Acid and Creatine are Associated with Cardiometabolic Risk Factors in Healthy Men and Women: A Cross-Sectional Study

**DOI:** 10.3390/nu10010087

**Published:** 2018-01-13

**Authors:** Sergej M. Ostojic, Milan Vranes, Davor Loncar, Natasa Zenic, Damir Sekulic

**Affiliations:** 1 Faculty of Sport and Physical Education, University of Novi Sad, 21000 Novi Sad, Serbia; 2 School of Medicine, University of Belgrade, 11000 Belgrade, Serbia; 3 Faculty of Sciences, University of Novi Sad, 21000 Novi Sad, Serbia; milan.vranes@dh.uns.ac.rs; 4 Institute SUPERLAB, 11000 Belgrade, Serbia; dloncar@superlab.rs; 5 Faculty of Kinesiology, University of Split, 21000 Split, Croatia; natasa@kifst.hr (N.Z.); dado@kifst.hr (D.S.)

**Keywords:** guanidinoacetic acid, creatine, cardiometabolic risk, homocysteine, overweight

## Abstract

Guanidinoacetic acid (GAA) conversion to creatine is thought to be involved in cardiometabolic disturbances through its role in biological methylation and insulin secretion. We evaluated the association of serum GAA and creatine with cardiometabolic risk factors in a cohort of 151 apparently healthy adults (82 women and 69 men) aged 18–63 years. Serum levels of GAA and creatine were measured with liquid chromatography-tandem mass spectrometry. A multiple linear regression model adjusted for age and sex was employed to examine the relationship of serum GAA and creatine with cardiometabolic risk factors. Higher GAA levels were associated with an unfavorable cardiometabolic risk profile (higher insulin, higher total homocysteine, and higher body fat percentage), while having elevated serum creatine levels (≥31.1 µmol/L) was associated with being overweight (body mass index ≥ 25.0 kg/m). The results from our study suggest a possible role of the GAA–creatine axis in the pathogenesis of cardiovascular and metabolic diseases.

## 1. Introduction

Guanidinoacetic acid (GAA) and creatine are natural amino acid derivatives that are heavily involved in cellular energy metabolism [1,2,3]. Much attention has been given recently to the potential role of these energy-related compounds in cardiometabolic diseases. For example, the high creatine kinase (CK) phenotype has been found in hypertension- and obesity-prone patients [4,5,6], and high CK activity has been reported to lead to dysfunctional vascular contractility and insulin resistance [6]. In addition, the biosynthesis of creatine from GAA is considered critical for methyl group consumption in humans [7], with altered methylation and consequent homocysteine production suggested as additional factors for cardiovascular disease risk [8,9]. Although the main focus has been on homocysteine metabolism, there have also been links between the GAA–creatine axis and classical cardiometabolic biomarkers, including insulin sensitivity and blood cholesterol [10]. In this study, we evaluated the associations of serum GAA and creatine with cardiometabolic risk factors—including body mass index (BMI), lipid profiles, high-sensitive C-reactive protein (CRP), glucose, and insulin—in apparently healthy men and women. A prespecified hypothesis suggested a negative correlation between an unfavorable cardiometabolic risk profile (including higher fasting insulin, higher total homocysteine, and higher body fat percentage) and serum levels of GAA and creatine. 

## 2. Materials and Methods

### 2.1. Participants

This study involved a secondary analysis of a subset of the Diet and Physical Activity for Health Initiative (DiPAH) cohort (ClinicalTrials.gov ID: NCT01958333) that underwent cardiometabolic risk assessment and had archived serum. The DiPAH was started in 2008 as a long-term, nationally recognized health study that is focused on strategies for the prevention of chronic diseases in the Serbian population. The present study was a cross-sectional study designed to identify relationships between energy-related biomarkers of micronutritional status and cardiometabolic disease risk factors in healthy adults. Participants were selected to join the study on the basis of the following criteria: (a) previous participation in DiPAH trials; (b) age between 18 and 65 years; (c) no medical conditions that would limit the successful completion of the protocol; and (d) current residence in Serbia. Pregnant women, individuals taking dietary supplements, and individuals engaged in a programmed exercise regimen were excluded from the study. Of 11069 DiPAH subjects initially considered, 151 participants (82 women and 69 men) were eligible and consented to participate in the current study. The minimal sample size (*n* = 120) was calculated according to power analysis for correlation point biserial model, with the effects size set at 0.25, a two-tail alpha level of 0.05, and a study power of 0.80 (G-Power 3, Heinrich Heine University Düsseldorf, Düsseldorf, Germany). The study was conducted according to the guidelines laid down in the Declaration of Helsinki, and all procedures were approved by the local IRB at the University of Novi Sad. 

### 2.2. Experimental Procedures

Blood samples after an overnight fast of 10 h and anthropometric measures were obtained by trained research staff. A fasting sample of venous blood was immediately centrifuged, with serum stored in a freezer at −80 °C, and the sample was analyzed for specific biomarkers after study completion. Serum GAA and creatine were measured by liquid chromatography-tandem mass spectrometry (SCIEX LC-MS/MS 5500QTRAP, AB Sciex Ltd., Concord, ON, Canada). Lipid profiles and glucose were analyzed by standard enzymatic methods with an automatic analyzer (Hitachi 704, Tokyo, Japan), while C-reactive protein was measured with a high sensitivity immunoturbidimetric assay (Hitachi 912, Tokyo, Japan). Insulin was evaluated with the CLIA method by automated chemiluminescence. Total serum homocysteine was determined with a chemiluminescent immuno-assay method using a chemistry analyzer (DPC Immulite 2000, Siemens, Berlin, Germany). Height was measured by a stadiometer (Seca 213, Hamburg, Germany), while weight and body fat percentage were measured by a bioelectrical impedance analyzer (Omron BF 511, Kyoto, Japan). Body mass index (BMI) was calculated as weight in kilograms divided by the square of height in meters, and participants were categorized as non-overweight (BMI < 25.0 kg/m^2^) or overweight (BMI ≥ 25.0 kg/m^2^). All participants were assessed in underwear after voiding, and the same trained technician did the anthropometric assessment in aim to minimize the testing error.

### 2.3. Statistical Analyses

Data are presented as the means ± standard deviation (SD) or frequencies (%). A multiple linear regression model with a stepwise method (adjusted for age and sex) was used to examine the relationships between serum GAA and serum creatine levels, and cardiometabolic risk factors. Multivariate-adjusted odds ratios for overweight participants were calculated across quartile categories of GAA and creatine, with a 95% confidence interval (CI) presented and a linear trend assessed across quartiles. Serum GAA and creatine were compared across sex and BMI categories using a two-tailed independent *t*-test. The significance level was set at *p* ≤ 0.05. The data were analyzed using the statistical package SPSS, version 21.0 (SPSS Inc., Chicago, IL, USA).

## 3. Results

**Figure 1:**
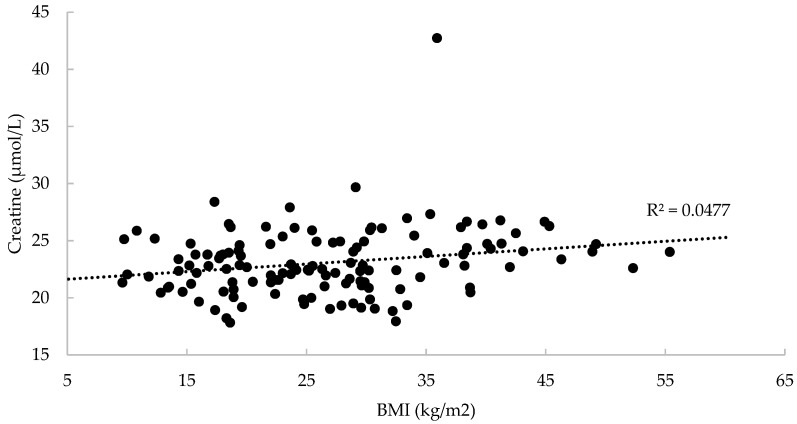
Correlation between serum creatine and body mass index (BMI) (*n* = 151).

Baseline characteristics of the study sample are depicted in Table 1. The mean ± SD age was 25.5 ± 6.9 years, with both genders were almost equally represented (54.3% women), and ~1 in 4 participants was overweight. The mean serum creatine levels were greater by 5.1 µmol/L in men than in women (95% CI 2.2–8.0 µmol/L; *p* = 0.001), whereas creatine concentrations were lower by 3.5 µmol/L in normal weight vs. overweight participants (95% CI 0.1–6.9 µmol/L; *p* = 0.05) (Table 2). A positive correlation has been found between serum creatine and BMI (*r* = 0.22, *p* = 0.01) (Figure 1). 

The odds of being overweight were similar across quartiles of serum GAA concentrations after controlling for age and sex (Table 3). A significant trend for greater odds of being overweight has been reported in participants with serum creatine levels in the fourth quartile than in the first three quartiles (*p* = 0.04).

Multiple regression analysis revealed that our model as a whole explained 60.3% of the variance in cardiometabolic risk biomarkers when serum GAA was included as a predictor variable (*R* = 0.78; adjusted *R*^2^ = 0.39, standard error of the estimate = 0.89) and 18.8% of the variance when serum creatine was included as a predictor variable (*R* = 0.43; adjusted *R*^2^ = 1.84, standard error of the estimate = 16.6), with gender and age accounted as control variables. There were no significant relationships between blood lipids and serum GAA or serum creatine concentrations when adjusted for age and sex (Table 4). Glucose was inversely associated with serum GAA and serum creatine levels, while insulin was positively associated with GAA levels only. Serum total homocysteine and body fat percentage were positively associated with serum GAA and serum creatine concentrations. Neither C-reactive protein nor body mass index were associated with serum GAA and serum creatine levels. In addition, serum creatine was positively associated with serum GAA concentrations when adjusted for age and sex (*β* = 0.32; *p* < 0.0001). 

## 4. Discussion

In a cohort of apparently healthy adults, serum GAA, and creatine concentrations had significant associations with several cardiometabolic risk factors. Higher GAA levels were associated with an unfavorable cardiometabolic risk profile (including higher fasting insulin, higher total homocysteine, and higher body fat percentage), while participants with elevated serum creatine levels (≥31.1 µmol/L) had greater odds of being overweight. This suggests a possible role of GAA–creatine axis in the pathogenesis of cardiovascular and metabolic disease.

Our findings were consistent with previous studies reporting a link between GAA and cardiometabolic disorders. Elevated serum GAA levels were associated with higher tHcy levels [11] and insulin hypersecretion [12], unfavorable risk factors for cardiometabolic diseases. We found that tHcy is a very strong predictor (*β* = 0.30, *p* < 0.001) and so it could be that tHcys is the root issue, either as an independent factor or through affecting arginine availability, since GAA is synthesized from arginine [1]. Hypothetically, enhanced GAA synthesis might restrain the availability of arginine, an amino acid that has been shown to strongly affect the risk factors of cardiovascular diseases in humans [13]. However, previous reports about the associations between serum creatine and cardiovascular and metabolic risk factors are not in line with our results. In several small-scale studies, higher serum creatine levels (as provoked by oral intake) were associated with a favorable risk-factor profile, including lower tHcy [14], reduced total cholesterol [15], or improved insulin sensitivity [16]. In contrast, a number of studies found no significant relationships between creatine alone and different cardiometabolic markers (for review see [10]), and a recent large study (*n* = 622) suggested no association between serum creatine and tHcy levels in apparently healthy men and women [17]. The conflict between our study and results from previous creatine studies may have been partially due to whether the analyses were based on serum levels or dietary intakes of GAA and creatine. The above studies typically linked creatine levels with cardiometabolic risk factors during an exercise program, an intervention known to reduce cardiometabolic risks per se [18], while we evaluated a diverse population not currently involved in an exercise program or dietary intervention. Our findings are in accordance with previous trials reporting an association between higher BMI and higher serum creatinine, an end-product of creatine metabolism [19], suggesting a link between the GAA–creatine axis and risk of being overweight. Hypothetically, GAA overload (as indicated by higher levels of serum GAA) may perturb creatine metabolism, thereby resulting in enhanced creatine synthesis that accounts for an equivalent proportion of tHcy production and cardiometabolic burden. We found that serum GAA is positively associated with insulin levels (*β* = 0.53), while no such relationship was found between serum creatine and insulin levels. Having higher GAA concentrations appears to be accompanied by elevated levels of insulin circulating in the blood, suggesting a possible link between excess GAA and hyperinsulinemia, a well-known cardiometabolic risk factor. Our results corroborate a previous in vitro study showing that GAA stimulates insulin release more potently than creatine, by triggering insulin secretion via kinase-sensitive mechanisms in pancreatic islets cells [12]. However, before putting forward serum GAA as a proxy for insulin release, additional studies are highly warranted to analyze GAA dynamics in populations with normal and impaired insulin secretion and action. In addition, creatine synthesis from GAA might affect cardiovascular risk through depletion of arginine, a precursor of creatine and a key source of nitric oxide (NO) [20]. NO plays an important role in the protection against the onset and progression of cardiovascular disease [21], and creatine overload may induce disturbances in NO bioavailability leading to a loss of the cardioprotective actions and in some populations may increase disease progression [22]. Therefore, synchronized monitoring of GAA-creatine and arginine-NO axes in future studies should provide more mechanistic evidence by which creatine synthesis affects cardiovascular risk.

Baseline levels of GAA and creatine were in accordance with previous studies reporting reference values for these two compounds [23,24,25], and gender-related differences for serum creatine found in the present study could be attributed to the effects of testosterone and estrogen on creatine synthesis and transport. The CK activity, which regulates the use and consumption of creatine, is found to be sex-specific [26]. Gender differences in muscle mass and responses to downstream signaling pathways in the skeletal muscle might also affect creatine utilization [27]. In particular, sex hormones appear to differently affect creatine transporter (SLC6A8) expression, with SLC6A8 inhibited by estrogens and stimulated by testosterone [28]. This has been suggested to provoke a higher leakage of body creatine in the urine of healthy women [25], with perhaps less creatine retained in the blood, as we found in the present study. In addition, we found that overweight adults had higher creatine concentrations (by 13.6% on average) compared to adults of a normal weight, and the odds ratio of being overweight was 3.26-fold higher in participants with a serum creatine ≥ 31.1 µmol/L. Previous studies confirm our findings, with a positive association having been reported between serum creatinine and BMI in healthy men and women [19,29]. The origin of creatinine from creatine may explain this correlation and the higher concentrations in men than women [30]. However, the strong positive correlation found between serum creatine levels (also GAA) and body fat percentage in our cohort remains puzzling. This perhaps means that circulating values of these compounds are related not only to body size but also to body composition and other (patho) physiological mechanisms that need to be revealed.

The major limitation of the present study is its cross-sectional design that prevented causal conclusions between the GAA-creatine axis and cardiometabolic risk factors. Second, we recruited a specific cohort of participants (predominantly young normal-weight adults, with no major biochemical disturbances) that limited the interpretation of our results to a healthy population while being unable to generalize the results to patients with cardiometabolic diseases. Third, we used only a finite compendium of biochemical variables to understand a more complete picture of GAA-creatine metabolism. Fourth, no link has been established between GAA and creatine levels and clinical indicators of cardiometabolic diseases, including markers of vascular pathology or metabolic overload. Finally, no skeletal muscle mass was profiled and accounted for in the regression analysis. Since skeletal muscle is a major site of insulin resistance [31], with creatine utilization found to be different among type I and type II muscle fibers [32], controlling for muscle mass in future studies could help to better address the link between biomarkers of GAA-creatine metabolism and cardiometabolic risk.

## 5. Conclusions

In conclusion, our results that GAA and creatine are associated with specific cardiometabolic risk factors (e.g., fasting insulin, total homocysteine, and body fat percentage) suggest a possible role of these energy-related compounds in the pathogenesis of cardiovascular and metabolic disease. The results from our study may help to better understand the metabolic aspects of these disorders, and suggest further evaluation of serum GAA and creatine applicability in the identification of adults with cardiometabolic disease risks.

## Figures and Tables

**Table 1:** Sample characteristics (*n* = 151). Values are mean ± standard deviation (SD).

Variable	Value	Range
Age (years)	25.5 ± 6.9	18.0–63.0
Sex, F (%)	54.3	-
BMI (kg/m^2^)	22.9 ± 2.9	17.6–42.7
Overweight ^1^ (%)	25.8	-
Body fat (%)	21.3 ± 7.5	7.4–47.6
Total cholesterol (mmol/L)	4.7 ± 0.7	3.4–7.2
LDL cholesterol (mmol/L)	3.1 ± 0.7	1.4–5.1
HDL cholesterol (mmol/L)	1.4 ± 0.3	0.6–2.7
Triglycerides (mmol/L)	1.1 ± 0.6	0.4–5.8
Glucose (mmol/L)	4.7 ± 0.8	2.2–6.4
Insulin (IU/L)	8.8 ± 6.2	3.9–29.7
tHcy (µmol/L)	8.5 ± 1.9	4.4–12.5
CRP (mmol/L)	0.002 ± 0.001	0.001–0.004
GAA (µmol/L)	2.6 ± 0.7	0.9–4.5
Creatine (µmol/L)	26.6 ± 9.3	9.6–55.4

BMI—body mass index; tHcy—total homocysteine; CRP—C-reactive protein; GAA—guanidinoacetic acid; F—female. ^1^ BMI ≥ 25.0 kg/m^2^.

**Table 2:** Serum levels of guanidinoacetic acid (GAA) and creatine. Values are mean ± SD.

	GAA (µmol/L)	Creatine (µmol/L)
Sex categories		
Men (*n* = 69)	2.6 ± 0.7	29.4 ± 10.4
Women (*n* = 82)	2.6 ± 0.7	24.3 ± 7.7 *
BMI categories		
Normal weight (*n* = 112)	2.6 ± 0.7	25.7 ± 8.9
Overweight (*n* = 39)	2.6 ± 0.8	29.2 ± 10.1 *

An asterisk (*) indicates a significant difference between categories at *p* < 0.05 (2-tailed independent *t*-test).

**Table 3:** Multivariate-adjusted odds ratios (95% CI in parentheses) for overweight (BMI ≥ 25.0 kg/m^2^) across quartile categories of serum guanidinoacetic acid (GAA) and creatine.

GAA					Creatine				
Quartile with µmol/L Range					Quartile with µmol/L Range				
I	II	III	IV	*p* trend	I	II	III	IV	*p* trend
0.90–2.19	2.20–2.79	2.80–2.99	3.00–4.50		9.6–18.9	19.0–26.2	26.3–31.0	31.1–54.4	
1.00	0.57	0.68	0.84	0.66	1.00	1.18	0.97	3.26	0.04
(--)	(0.19 to 1.69)	(0.24 to 1.94)	(0.31 to 2.31)		(--)	(0.38 to 3.68)	(0.30 to 3.09)	(1.14 to 9.32)	

**Table 4:** Multiple linear regression coefficients for cardiometabolic risk factors in relation to serum levels of guanidinoacetic acid (GAA) and creatine (*n* = 151).

	GAA		Creatine	
	*β*	*p*	*β*	*p*
Blood lipids				
Total cholesterol	−0.11	0.20	0.07	0.41
LDL cholesterol	−0.05	0.54	0.05	0.54
HDL cholesterol	0.08	0.35	0.13	0.12
Triglycerides	0.16	0.08	0.11	0.19
Glucose	−0.28	0.00	−0.26	0.00
Insulin	0.53	0.03	−0.17	0.57
tHcy	0.30	0.00	0.17	0.05
CRP	0.36	0.16	−0.26	0.38
Body mass index	0.02	0.87	0.11	0.23
BFP	0.32	0.03	0.37	0.01

The multiple linear regression model was adjusted for age and sex. tHcy—total homocysteine; CRP—C-reactive protein; BFP—body fat percentage.

## Abbreviations

BFP	Body fat percentage
BMI	Body mass index
CK	Creatine kinase
CRP	C-reactive protein
DiPAH	Diet and Physical Activity for Health Initiative study
GAA	Guanidinoacetic acid
SLC6A8	Creatine transporter
tHcy	Total homocysteine

## References

[1] Wyss M., Kaddurah-Daouk R. (2000). Creatine and creatinine metabolism. Physiol. Rev..

[2] Brosnan J.T., Brosnan M.E. (2007). Creatine: Endogenous metabolite, dietary, and therapeutic supplement. Ann. Rev. Nutr..

[3] Ostojic S.M. (2015). Cellular bioenergetics of guanidinoacetic acid: The role of mitochondria. J. Bioenerg. Biomembr..

[4] Brewster L.M., Mairuhu G., Bindraban N.R., Koopmans R.P., Clark J.F., van Montfrans G.A. (2006). Creatine kinase activity is associated with blood pressure. Circulation.

[5] Haan Y.C., van Montfrans G.A., Brewster L.M. (2015). The high creatine kinase phenotype is hypertension- and obesity-prone. J. Clin. Hypertens..

[6] Johnsen S.H., Lilleng H., Bekkelund S.I. (2014). Creatine kinase as predictor of blood pressure and hypertension. Is it all about body mass index? A follow-up study of 250 patients. J. Clin. Hypertens..

[7] Stead L.M., Brosnan J.T., Brosnan M.E., Vance D.E., Jacobs R.L. (2006). Is it time to reevaluate methyl balance in humans?. Am. J. Clin. Nutr..

[8] Glier M.B., Green T.J., Devlin A.M. (2014). Methyl nutrients, DNA methylation, and cardiovascular disease. Mol. Nutr. Food. Res..

[9] Wald D.S., Law M., Morris J.K. (2002). Homocysteine and cardiovascular disease: Evidence on causality from a meta-analysis. BMJ.

[10] Pinto C.L., Botelho P.B., Pimentel G.D., Campos-Ferraz P.L., Mota J.F. (2016). Creatine supplementation and glycemic control: A systematic review. Amino Acids.

[11] Ostojic S.M., Niess B., Stojanovic M., Obrenovic M. (2013). Co-administration of methyl donors along with guanidinoacetic acid reduces the incidence of hyperhomocysteinaemia compared with guanidinoacetic acid administration alone. Br. J. Nutr..

[12] Alsever R.N., Georg R.H., Sussman K.E. (1970). Stimulation of insulin secretion by guanidinoacetic acid and other guanidine derivatives. Endocrinology.

[13] Nittynen L., Nurminen M.L., Korpela R., Vapaatalo H. (1999). Role of arginine, taurine and homocysteine in cardiovascular diseases. Ann. Med..

[14] Korzun W.J. (2004). Oral creatine supplements lower plasma homocysteine concentrations in humans. Clin. Lab. Sci..

[15] Earnest C.P., Almada A.L., Mitchell T.L. (1996). High-performance capillary electrophoresis-pure creatine monohydrate reduces blood lipids in men and women. Clin. Sci..

[16] Gualano B., De Salles Painneli V., Roschel H., Artioli G.G., Neves M., De Sá Pinto A.L., Da Silva M.E., Cunha M.R., Otaduy M.C., Leite Cda C. (2011). Creatine in type 2 diabetes: A randomized, double-blind, placebo-controlled trial. Med. Sci. Sports Exerc..

[17] Peters B.A., Hall M.N., Liu X., Parvez F., Siddique A.B., Shahriar H., Uddin M.N., Islam T., Ilievski V., Graziano J.H. (2015). Low-dose creatine supplementation lowers plasma guanidinoacetate, but not plasma homocysteine, in a double-blind, randomized, placebo-controlled trial. J. Nutr..

[18] Ekelund U., Luan J., Sherar L.B., Esliger D.W., Griew P., Cooper A. (2012). Moderate to vigorous physical activity and sedentary time and cardiometabolic risk factors in children and adolescents. JAMA.

[19] Banfi G., Del Fabbro M. (2006). Relation between serum creatinine and body mass index in elite athletes of different sport disciplines. Br. J. Sports Med..

[20] Karamat F.A., van Montfrans G.A., Brewster L.M. (2015). Creatine synthesis demands the majority of the bioavailable L-arginine. J. Hypertens..

[21] Naseem K.M. (2005). The role of nitric oxide in cardiovascular diseases. Mol. Aspects Med..

[22] Brewster L.M., Seedat Y.K. (2013). Why do hypertensive patients of African ancestry respond better to calcium blockers and diuretics than to ACE inhibitors and β-adrenergic blockers? A systematic review. BMC Med..

[23] Carducci C., Birarelli M., Leuzzi V., Carducci C., Battini R., Cioni G., Antonozzi I. (2002). Guanidinoacetate and creatine plus creatinine assessment in physiologic fluids: An effective diagnostic tool for the biochemical diagnosis of arginine:glycine amidinotransferase and guanidinoacetate methyltransferase deficiencies. Clin. Chem..

[24] Almeida L.S., Verhoeven N.M., Roos B., Valongo C., Cardoso M.L., Vilarinho L., Salomons G.S., Jakobs C. (2004). Creatine and guanidinoacetate: Diagnostic markers for inborn errors in creatine biosynthesis and transport. Mol. Genet. Metab..

[25] Curt M.J.-C., Cheillan D., Briand G., Salomons G.S., Mention-Mulliez K., Dobbelaere D., Cuisset J.-M., Lion-François L., Des Portes V., Chabli A. (2013). Creatine and guanidinoacetate reference values in a French population. Mol. Genet. Metab..

[26] Sömjen D., Lundgren S., Kaye A.M. (1997). Sex and depot-specific stimulation of creatine kinase B in rat adipose tissues by gonadal steroids. J. Steroid Biochem. Mol. Biol..

[27] Tarnopolsky M.A. (2000). Gender differences in metabolism; nutrition and supplements. J. Sci. Med. Sport.

[28] Shojaiefard M., Christie D.L., Lang F. (2005). Stimulation of the creatine transporter SLC6A8 by the protein kinases SGK1 and SGK3. Biochem. Biophys. Res. Commun..

[29] Vikse B.E., Vollset S.E., Tell G.S., Refsum H., Iversen B.M. (2004). Distribution and determinants of serum creatinine in the general population: The Hordaland Health Study. Scand. J. Clin. Lab. Investig..

[30] Perrone R.D., Madias N.E., Levey A.S. (1992). Serum creatinine as an index of renal function: New insights into old concepts. Clin. Chem..

[31] Julius S., Gudbrandsson T., Jamerson K., Tariq Shahab S., Andersson O. (1991). The hemodynamic link between insulin resistance and hypertension. J. Hypertens..

[32] Lillioja S., Young A.A., Culter C.L., Ivy J.L., Abbott W.G., Zawadzki J.K., Yki-Järvinen H., Christin L., Secomb T.W., Bogardus C. (1987). Skeletal muscle capillary density and fiber type are possible determinants of in vivo insulin resistance in man. J. Clin. Investig..

